# Knowledge, attitudes, and practices regarding blood donation among rural adults aged 18–59 years in Bihar, India: A community-based cross-sectional study

**DOI:** 10.1177/20503121251387217

**Published:** 2026-02-05

**Authors:** Manisha Verma, Shreyas Patil, Rajath Rao, Bijaya Nanda Naik, Santosh Kumar Nirala, Mohit Bhardwaj

**Affiliations:** 1 Department of Community Medicine, NAMO Medical Education and Research Centre, Silvassa, Dadra and Nagar Haveli, India; 2 Department of Community Medicine and Family Medicine, All India Institute of Medical Sciences Bibinagar, Telangana, India; 3 Department of Community Medicine, Kasturba Medical College Mangalore, Manipal Academy of Higher Education, Manipal, India; 4 Department of Community and Family Medicine, All India Institute of Medical Sciences Patna, Bihar, India

**Keywords:** Blood donation, blood group, pain perception, barriers, perception, rural India, Bihar

## Abstract

**Objectives:**

The World Health Organization (WHO) advocates for voluntary blood donation by healthy donors to ensure the availability of safe blood. India faces a significant gap between blood demand and supply for various acute and chronic conditions. This study assesses blood donation knowledge, attitudes, and practices, as well as the barriers affecting the willingness to donate blood and predictors of good knowledge and favorable attitudes toward blood donation among the rural adult population in Bihar.

**Methods:**

A community-based cross-sectional study was conducted from January to June 2023 in the Naubatpur block of Patna district, Bihar, involving 500 adults aged 18–59 years via a multistage sampling technique for the enrollment of participants. Data were collected via a pretested semistructured questionnaire. Descriptive and inferential statistics were used, and multivariable logistic regression was used to identify predictors of knowledge and attitude scores.

**Results:**

Approximately 67.4% of the respondents were aware of blood donation, while 39.8% were aware of their blood group. Only 10.8% had donated blood, with 58.5% having donated only once. Nearly 29.08% had good knowledge regarding blood donation. Major barriers included no specific reason, perceived pain, and a fear of needles.

**Conclusion:**

In our study, nearly two out of three participants were aware of blood donation, but hardly one out of three was aware of their blood group. Only slightly more than one-fourth had good knowledge and a favorable attitude toward blood donation, while only one out of 10 had donated blood in the past. Despite good knowledge about blood donation, actual practices were low among the study population. Educational level significantly influences awareness and attitudes. Health education and periodic awareness programs are essential to dispel myths and promote voluntary blood donation in rural areas.

## Introduction

Voluntary blood donation in India began in 1942 during World War II, when blood was vital in saving the lives of the injured and infirm. During this period, the donors were mainly government employees and those from the Anglo–Indian fraternity who donated blood for a generous cause.^1^ However, the number of voluntary donors declined after the war, and the donors had to be paid for their blood.^2^ According to the World Health Organization (WHO), the safest category of blood donors is those who donate voluntarily without remuneration from low-risk populations.^3^ There is a large gap between the demand for and supply of blood in developing countries, particularly India, due to the high prevalence of nutritional anemia.^4^ It is estimated that India needs an equivalent of 85 donations per 1000 eligible people, which was found to be 31 donations per 1000 eligible people in 2018. This enormous deficit must be bridged through collaborative, multisectoral efforts, as donated blood is pivotal in saving the lives of those who require large volumes of blood in medical emergencies.^5,6^ Blood donors are motivated by reasons such as altruism, humanitarian inclinations, personal or family credit, social pressure, replacement, and reward. On the other hand, fear of needles, contracting an infection, and other adverse effects, including medical problems, dissuade donors.^7,8^ Previous studies have noted that blood donation practices can be poor despite good knowledge and favorable attitudes.^9–13^ Gender disparities in blood donation have also been observed, with females being less likely to donate.^14,15^

In India, 84.3% of blood units collected through voluntary blood donation at the National AIDS Control Organization (NACO) supported blood banks in 2012.^16^ To date, recruiting volunteers for blood donation remains a major challenge, as evidenced by the 12000 deaths that occur each day in India due to a dearth of safe and timely donated blood.^17,18^ Moreover, systemic deficiencies have resulted in a situation where several Indian districts do not have a single blood bank.^16,19^ Evidence indicates that approximately 230 million surgeries, 331 million cancer-related procedures, and 10 million pregnancy-related procedures occur in India every year, resulting in a significant demand for blood.^20,21^ Therefore, it is paramount to take a closer look at the blood donation practices prevalent in the community and the factors affecting them. We aimed to determine the knowledge, attitudes, and practices related to blood donation; to estimate the prevalence of blood donation in the past; to determine the predictors of good knowledge and favorable attitudes toward blood donation; and to identify the barriers that affect blood donation practices among nondonors in the rural adult population of Bihar. To the best of our knowledge, this is the first study of its kind in the Indian state of Bihar.

## Methodology

### Study design and duration

This was a community-based cross-sectional study, and data collection took place over six months between January and June 2023. The total duration of the study, including the preparation/preliminary phase of data analysis, was 1 year.

### Study setting

The study was conducted in the Naubatpur block, the rural field practice area of the Department of Community and Family Medicine of the Institute of National Importance (INI) in the Patna district of Bihar, India. The Naubatpur block is one of the rural blocks of the 23 existing administrative blocks of the Patna district and is located 15 km north of the INI. The rural field practice area of this INI caters to the health needs of 12 villages out of 100 villages and 19 gram panchayats in this block. According to the 2011 census, Naubatpur has a population of approximately 1,78,583, covering 30,211 rural households spread over 151.12 km^2^ of rural area.^22,23^

### Study population

The study included all the adults (⩾ 18 years to 59 years) residing in the rural field practice area of the INI and who had been living in the village for at least 1 year preceding the date of the interview. The participants who did not provide written informed consent to participate in the study were excluded from the study.

### Sample size and sampling technique

The representative target sample size was calculated to be 500 to achieve the study objectives with an open-source sample size calculator, Statulator. The sample size was calculated on the basis of the prevalence of blood donation practices. The sample size calculation resulted in 469 participants, rounding to 500 for a proportion of blood donations in the past among rural adults of 17.5% using a relative precision of 20% and a confidence level of 95%.^10,24^

A multistage sampling technique was adopted to enroll the participants. In the first stage, all the villages lying within a 5 km radius of the Rural Health Training Center (RHTC) of the INI were listed. Five villages out of the 21 listed villages were chosen via simple random sampling. In the second stage, 100 houses from each village were chosen via a systematic random sampling method. With a sampling interval of 5, every fifth house out of an average household size of 500 was chosen for the study. In the third stage, household members were assessed, and one eligible member meeting the inclusion and exclusion criteria was chosen for the study. If a household had more than one eligible participant, one of them was selected via the lottery method.

### Study tool and procedure

A predesigned, semistructured, pretested questionnaire was used to collect information from the participants. The questionnaire was developed in English and later translated into the local language (Hindi) and back-translated to English with the help of medical-social workers (MSWs) in the department. The forward-backward translations were performed as per the WHO guidelines for language translation. The study tool underwent face validation by two independent experts from the fields of public health and transfusion medicine. The content validity index (CVI) was calculated after the independent rating regarding clarity, ambiguity, and relevance by three experts other than those who were involved in facial validation, and the CVI-S was found to be 0.9, indicating a strong level of agreement by the experts. Pretesting was performed among 50 participants (10% of the sample size) who were not a part of this study and were coming to the RHTC of the INI. The internal reliability, as depicted by Cronbach’s alpha, was found to be 0.8 (good internal consistency). The study tool was administered via face‒to‒face interviews in the local language. The study tool consisted of the following sections: Section A collected the sociodemographic details of the participants’ age, gender, residence, education, occupation, and blood group. Section B consisted of questions related to knowledge regarding blood donation, such as who can donate blood, the amount of blood that should be donated during one session, the minimum age of donation, body weight, frequency, and the minimum interval between two donations. Section C consisted of items related to the attitudes of the respondents toward blood donation, whether they perceive blood donation to be an important act, whether blood donation saves lives, and whether they accept blood donation from others. Section D consisted of items related to practices regarding blood donation. This section applied only to those respondents who had donated blood in the past. Section E consisted of items related to barriers to blood donation. Certain prompts were included, such as “The hours are inconvenient”, “it would be painful”, and “I would feel dizzy and faint.” The items in the knowledge section were scored as follows: “1” for every right response and “0” for an incorrect response. Few items in the knowledge domain had multiple correct responses. In total, there were 10 items in the knowledge section, resulting in a score range of 0–16. A score greater than or equal to the 75th percentile was considered good knowledge related to blood donation. Similarly, questions related to attitudes were scored as ‘1’ or ‘0’ with a score ranging from 0 to 4. An overall score greater than or equal to the 75th percentile was considered a favorable attitude toward blood donation.

### Statistical analysis

The collected data were imported into MS Excel and analyzed via IBM SPSS V.22.0 (IBM Corp., Armonk, NY, USA). Descriptive analysis was performed to describe the sociodemographic details of the respondents. Categorical variables such as awareness of blood donation, attitudes toward blood donation, practices related to blood donation, and barriers to blood donation were expressed as frequencies and percentages. Scores for knowledge and attitudes related to blood donation were computed and categorized into “good or poor knowledge” and “favorable or unfavorable attitudes” on the basis of the 75th percentile score. The proportion of respondents with good knowledge and favorable attitudes was expressed as percentages with a 95% confidence interval (CI). A multivariable logistic regression analysis was performed to identify the predictors of good knowledge and favorable attitudes toward blood donation. Predictors of blood donation practices were not analyzed because of the small sample size of people who donated. Variables with a *p* value of <0.2 in the univariable analysis were included in the multivariable regression analysis model via the enter method. The crude odds ratio (COR) and adjusted odds ratio (AOR) with 95% CI were reported. All statistical significance was attributed to a *p* value <0.05.

### Ethical considerations

This study was approved by the Institutional Ethics Committee, All India Institute of Medical Sciences, Patna (AIIMS/Pat/IEC/2022/952 dated 13-10-2022). We adhered to the principles of ethics throughout the study, and thereafter, the study was conducted in accordance with the Helsinki Declaration of 1975 as revised in 2024. Written informed consent was obtained from the participants upon their willingness to be a part of the study. All the identifiers of the participants were removed from the master chart and deidentified, and the data were informed that they were purely for research purposes and would be available to researchers only.

The reporting of the study conforms to the STROBE guidelines.^25^

## Results

### Sociodemographic characteristics of the participants

Table 1 shows the sociodemographic characteristics of the study respondents. The mean (SD) age of the respondents was 35.54 (11.68) years. There was a slight male predominance (283, 56.60%) in the study population, and the vast majority were Hindu by religion (468, 93.60%). Approximately one-quarter (23.80%) of the respondents did not have formal education; more than half (60.40%) had education levels ranging from primary to high school; and the rest (15.8%) were graduates or above. Students formed the largest group (173, 35.74%), whereas unemployed individuals (28, 5.79%) constituted the smallest group. Almost three-fourths (348, 71.46%) belonged to extended families (Table 1).

**Table 1.** Sociodemographic details of the participants (*n* = 500).

Variables	Categories	*n* (%)
Age (in years) [mean (SD)]		35.54 (11.68)
Gender (*n* = 500)	Female	217 (43.40%)
	Male	283 (56.60%)
Religion (*n* = 500)	Hindu	468 (93.60%)
	Muslim	32 (6.40%)
Education (*n* = 500)	No formal education	119 (23.80%)
	Primary	112 (22.40%)
	Secondary	87 (17.40%)
	High school	103 (20.60%)
	Graduate and above	79 (15.80%)
Occupation (*n* = 484)	Unemployed	28 (5.79%)
	Student	173 (35.74%)
	Housewife	46 (9.50%)
	Salaried	117 (24.17%)
	Self-employed	120 (24.79%)
Ration card (*n* = 500)	No	223 (44.60%)
	Yes	277 (55.40%)
Type of family (*n* = 487)	Nuclear	139 (28.54%)
	Extended	348 (71.46%)

### Awareness and knowledge regarding blood donation

The details of the respondents’ awareness and knowledge regarding blood donation are depicted in Table 2. The proportion of individuals with good knowledge scores was estimated to be 29.08% (95% CI: 24.49%–34.14%). Nearly two-thirds [337, 67.40%] were aware of blood donation; however, only 199 (39.80%) individuals were aware of their blood group. The majority (304, 90.20%) responded correctly that any healthy adult could donate blood. However, awareness of the correct amount of blood donated during a session stood at 25.82%, whereas 48.66% were rightly aware of the minimum weight for donating blood, and 43.03% were correctly aware of the minimum interval between two blood donations. When asked about possible places for blood donation, government blood banks were found to be the most common response (67.06%), followed by blood donation camps (52.22%) and private blood banks (31.75%).

**Table 2.** Details regarding blood donation (*n* = 500).

Variables	Categories	n (%)
Awareness regarding their own blood group (*n* = 500)	Not aware	301 (60.20%)
	aware	199 (39.80%)
Ever heard about blood donation (*n* = 500)	No	163 (32.60%)
	Yes	337 (67.40%)
Who can donate blood? ^a^ (*n* = 337)	Any healthy adult^b^	304 (90.20%)
	Alcoholics	12 (3.56%)
	Pregnant women	4 (1.18%)
	PLHIV	0
	Don’t know	33 (9.79%)
Amount of blood to be donated during one session (*n* = 337)	Don’t know	205 (60.83%)
	<250 ml	45 (13.35%)
	250–500 ml^b^	87 (25.82%)
Minimum weight for a person to donate blood (*n* = 337)	Don’t know	164 (48.67%)
	< 45 kg	9 (2.67%)
	> 45 kg ^b^	164 (48.66)
Minimum interval between two blood donations (*n* = 337)	Don’t know	182 (54.00%)
	<3 months	10 (2.97%)
	⩾3 months ^b^	145 (43.03%)
Infections that can spread through blood donation^a^ (*n* = 324)	Don’t know	168 (51.85%)
	HIV/AIDS ^c^	109 (33.64%)
	Hepatitis B ^c^	27 (8.33%)
	Cancer	4 (1.23%)
	TB	2 (0.62%)
	Malaria ^c^	14 (4.32%)
	Syphilis^c^	1 (0.31%)
Legal age for blood donation (*n* = 337)	Don’t know	163 (48.37%)
	16 years	2 (0.6%)
	18 years	172 (51.04%)
Person with low hemoglobin donate blood (*n* = 337)	No ^b^	260 (77.15%)
	Yes	77 (22.85%)
People with any blood group can donate blood (*n* = 337)	No	83 (24.63%)
	Yes ^b^	254 (75.37%)

a Multiple response questions.

b Correct response.

c Multiple correct responses

More than half (51.85%) of the respondents were unaware of the infections that could be spread through blood donation. HIV/AIDS was the most common blood-borne infection (33.64%), followed by hepatitis B (8.33%) and malaria (4.32%). Slightly more than half of the respondents (51.04%) correctly stated that the minimum legal age for donating blood was 18 years, whereas three-fourths (254, 75.37%) were aware that people with any blood group could donate blood.

### Attitude toward blood donation

**Figure 1.**
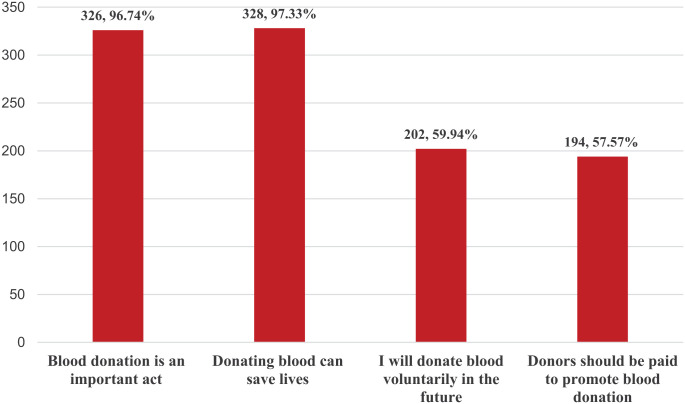
Attitudes toward blood donation among respondents (*n* = 337).

The responses related to attitudes toward blood donation are displayed in Figure 1. The proportion of individuals with a favorable attitude score toward blood donation was estimated to be 26.41% (95% CI: 21.99–31.36). Most respondents believed that blood donation is important (326, 96.74%) and that it can save lives (328, 97.33%). However, more than half (194, 57.57%) of the participants believed that donors should be paid to promote blood donation, and only three-fifths (202, 59.94%) stated that they would voluntarily donate blood in the future.

### Practices related to blood donation

Notably, only 54 (10.8%) respondents had donated blood in the past, and more than half (31, 58.49%) of them had donated on just one occasion. Approximately 22 (46.81%) respondents donated blood for their family and friends, whereas 25 (53.19%) donations were voluntary. More than four-fifths (44, 81.48%) of the respondents claimed to have received information before donating blood. The mode of information was solely verbal in 61.70% of the cases. Over four-fifths (45, 83.33%) of the respondents were asked health-related questions before donation, investigations were performed before donation in 72.22% of the cases, and refreshments following blood donation were provided in most cases (49, 90.74%). However, medical examination before donation was performed in less than half of the cases (25, 46.30%) (Table 3).

**Table 3.** Practices related to blood donation (*n* = 500).

Variables	Categories	n (%)
Donated blood in the past (*n* = 500)	No	446 (89.20%)
	Yes	54 (10.80%)
Frequency of donation (*n* = 53)	Donated just once	31 (58.49%)
	Donated more than once	22 (41.51%)
Reason behind donating blood (*n* = 47)	For family and friends	22 (46.81%)
	Voluntary donation	25 (53.19%)
Any health problems before donation (*n* = 54)	No	50 (92.59%)
	Yes	4 (7.41%)
Received any information before blood donation (*n* = 54)	No	10 (18.52%)
	Yes	44 (81.48%)
Mode of information (*n* = 47)	Only verbal	29 (61.70%)
	Verbal and printed	18 (38.30%)
Health-related questions asked before donation (*n* = 54)	No	9 (16.67%)
	Yes	45 (83.33%)
Investigations were done before donation (*n* = 54)	No	15 (27.78%)
	Yes	39 (72.22%)
Medical examination done before blood donation (*n* = 54)	No	29 (53.70%)
	Yes	25 (46.30%)
Refreshment provided after blood donation (*n* = 54)	No	5 (9.26%)
	Yes	49 (90.74%)

### Barriers to blood donation among the participants

**Figure 2.**
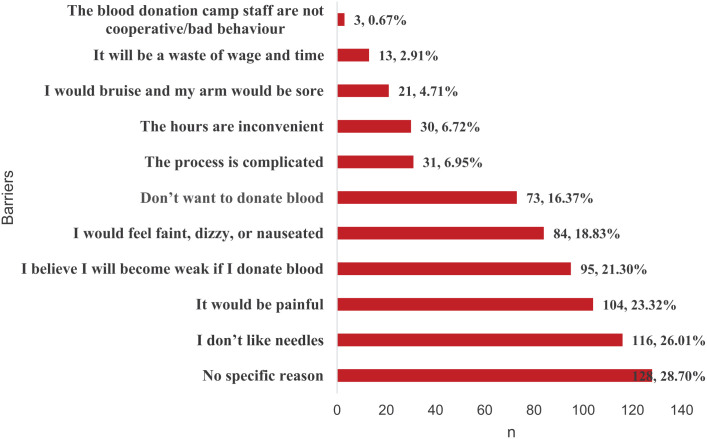
Barriers to blood donation among respondents (*n* = 446).

The leading barriers to blood donation among those who had never donated were found to be no specific reason (128, 28.70%), dislike for needles (116, 26.01%), and the perception that the process would be painful (104, 23.32%). Other barriers included the notions of becoming weak (21.3%), fainting, feeling dizzy or nauseated (18.8%) when donating blood. (Figure 2)

### Predictors of knowledge scores and attitudes scores regarding blood donation

Multivariate logistic regression analysis revealed that age [adjusted OR (AOR), 0.951; 95% CI, 0.918–0.984], an educational level of graduate and above [AOR, 5.082; 95% CI, 1.402–18.422], and a history of prior blood donation [AOR, 2.808; 95% CI, 1.367–5.769] were independent predictors of good knowledge regarding blood donation. Similarly, a history of prior blood donation [adjusted OR (AOR), 2.599; 95% CI, 1.232–5.480], possession of a ration card [AOR, 0.311; 95% CI, 0.171–0.563], and knowledge score [AOR, 1.214; 95% CI, 1.083–1.361] were found to be independent predictors of favorable attitudes toward blood donation. (Table 4)

**Table 4.** Predictors of knowledge and attitudes toward blood donation (*N* = 337).

Predictors	Good knowledge regarding blood donation		Favorable attitude toward blood donation	
	Crude OR (95% CI)	Adjusted OR (95% CI, p)	Crude OR (95% CI)	Adjusted OR (95% CI, p)
Age	0.958 (0.935–0.981)*	0.951 (0.918–0.984, 0.004)*	0.972 (0.949–0.995)*	0.973 (0.941–1.006)
Sex Female Male	1 1.992 (1.188–3.341)*	1 0.952 (0.345–2.628)	1 1.892 (1.111–3.22)*	1 2.021 (0.682–5.988)
Education Uneducated Primary school Secondary school High school Graduate and above	1 1.835 (0.555–6.059) 2.917 (0.904–9.407) 3.559 (1.140–11.107)* 10.937 (3.519–33.996)*	1 1.344 (0.383–4.716) 1.375 (0.386–4.899) 1.692 (0.483–5.926) 5.083 (1.402–18.422, 0.013)*	1 2.516 (0.671–9.42) 4.653 (1.279–16.92)* 5.473 (1.543–19.41)* 7.636 (2.145–27.17)*	1 1.645 (0.411–6.578) 2.127 (0.528–8.56) 2.065 (0.509–8.376) 1.640 (0.3702–7.264)
Occupation Unemployed Housewife Student Salaried Self-employed	1 0.716 (0.230–2.224) 2.040 (0.624–6.672) 2.630 (0.896–7.724) 1.275 (0.418–3.893)	1 1.400 (0.306–6.397) 1.366 (0.362–5.161) 3.019 (0.878–10.376) 2.607 (0.688–9.883)	1 0.561 (0.190–1.657) 1.011 (0.315–3.249) 1.376 (0.491–3.858) 0.814 (0.277–2.387)	1 0.804 (0.187–3.447) 0.319 (0.082–1.250) 0.712 (0.219–2.311) 0.544 (0.153–1.935)
Family Extended Nuclear	1 1.608 (0.981–2.637)	1 1.615 (0.914–2.854)	–	–
Blood group Not aware Aware	1 3.413 (2.046–5.694)*	1 1.347 (0.7135–2.543)	1 1.619 (0.989–2.65)	1 0.763 (0.389–1.492)
Past h/o blood donation No Yes	1 4.345 (2.373–7.957)*	1 2.808 (1.367–5.769, 0.005)*	1 2.950 (1.614–5.39)*	1 2.599 (1.232–5.480, 0.01)*
Ration card No Yes	–	–	1 0.409 (0.249–0.672)*	1 0.311 (0.171–0.563, <0.001)*
Knowledge score	–	–	1.221 (1.117–1.334)*	1.214 (1.083–1.361, <0.001)*

Nagelkerke’s *R*^2^ for knowledge model: 0.267, Nagelkerke’s *R*^2^ for attitude model: 0.232.

* Statistically significant

## Discussion

Unraveling the various factors that influence the awareness, perceptions, attitudes, and practices of rural communities with respect to blood donation is crucial. This can facilitate the effective execution of voluntary blood donation programs and ensure an adequate supply of safe blood for those in need. The sociodemographic profile of our study participants is in line with other studies in rural areas of India and elsewhere, reporting findings that parallel our study. ^9,10,13,26^ These observations are expected, as the study was conducted in a rural setting, where traditional family structures and low educational attainment are commonplace.

Awareness of the practice of blood donation was reported by over two-thirds (67.4%) of the respondents. This number is greater than that reported in community-based studies performed in Bukavu (39%) but lower than that reported in studies from Puducherry (79.5%), Vellore (90%), Hossana (92.18%), Harar (93.73%), and Debre Markos (100%). ^9,10,12,13,27,28^ Few studies from Gondar, Ethiopia (56.8%) or Bangladesh (68.6%) reported similar knowledge levels.^29,30^ These dissimilarities may be attributable to differences in the sociodemographic characteristics of these populations. Awareness regarding one’s blood group is desirable, as it facilitates receiving or donating blood in emergency situations. Moreover, it helps minimize medical errors and undue harm to recipients of blood transfusions. However, only 39.8% of the participants were aware of their blood group in the current study. This figure is lower than those of previous studies performed in Vellore (55%), Harar (60.12%), Hossana (67.3%) and Ebonyi (85.2%). ^9,13,27,31^ Regional disparities in health-seeking behaviors and health system-related factors could have resulted in these differences across studies. Higher awareness among individuals of their blood groups would be expected in regions with stronger health systems, with better diagnostic facilities, and where they are more likely to contact the formal health system.

Shortcomings in knowledge regarding other aspects of blood donation were also observed. The correct minimum weight for donating blood was known by 48.66% of the respondents in the present study. This percentage is higher than the percentages reported among adults of Ebonyi (4.3%) and Debre Markos (29.9%). However, this percentage was lower than that reported in studies performed in Adama (65.1%) and Hosanna (79.6%). ^11,12,27,31^ It was also noted that the minimum interval between two donations, that is, 3 months, was correctly stated by 43.03% of the respondents, which is consistent with similar studies conducted in Ethiopia. ^13,27^ However, observations from rural India in Vellore (69.2%) and Puducherry (14.8%) were found to diverge from our study. ^9,10^

The guidelines for blood donor selection in India stipulate that the minimum age for donation is 18 years.^32,33^ More than half (51.04%) of the participants were found to know this, compared with 63.4% reported by Kurup et al.^9^ Other population-based studies from Ethiopia, where the minimum age for blood donation is 18 years, also demonstrated a higher level of awareness, ranging from 62.2% to 72%. ^11,12,27^ A lack of knowledge can potentially hinder the development of favorable attitudes and adversely impact blood donation practices. Bridging these gaps through targeted health education programs is imperative to dispel myths and misconceptions and make individuals more receptive to the notion of donating blood.

The belief that blood donation was an important act and a life-saving act was agreed upon by 96.74% and 97.33% of the participants, respectively, which is consistent with community-based studies from Telangana and Ethiopia.^13,34^ Conversely, studies from Ethiopia and Nigeria report that attitudes toward blood donation are generally less supportive.^11,27,35^ Although the belief in its importance was high, only 59.94% of the participants in the present study agreed that they would voluntarily donate blood in the future. Willingness to voluntarily donate blood was found to be comparatively lower in Vellore (44%), Adama (39.8%), and Hosanna (46.9%) and higher in Lucknow (73.37%), Puducherry (75%), Debre Markos (73.96%), Harar (81. 7%) and Hyderabad (95.8%).^9–13,27,34,36^ These attitudinal changes across and within regions might stem from the multitude of social and cultural differences. Studies of a more qualitative nature would help in drawing deeper insights into these differences. These results could be utilized to design effective, regionalized social and behavioral change communication (SBCC) strategies to bring about desirable changes in attitudes. In contrast, almost half (42.3%) of the participants agreed that donors should be paid to promote blood donation. Remunerated blood donation is outlawed in India and is subject to various ethical, legal, and safety concerns. Nevertheless, context-specific nonmonetary incentives may be explored to improve blood donation, as deliberated in previous studies.^37,38^

Although the study participants were aware of and supported the idea of blood donation, this had failed to materialize into actual practice, as only 10.8% had donated in the past. Poor practices against the backdrop of good awareness and favorable attitudes are a recurring theme across several contexts.^9–13^ The National Blood Policy 2007 envisions the gradual phasing out of replacement blood donations, a move toward achieving 100% voluntary blood donations. Nevertheless, 46.81% of the donors in this study had donated to either friends or family. Replacement donations have also been commonly observed in similar settings. ^10,11,39^ Measures that improve voluntary nonremunerated blood donation, in turn curbing replacement donations, are thus necessary to realize the policy’s vision. ^40^

The barriers to blood donation in our study are substantiated by other studies.^10,11,33,34,36,41,42^ Several of these barriers are the product of myths and misconceptions related to blood donation and can be remedied by simple health education strategies. Additionally, gender itself is a barrier. Compared with males, females report greater fear of needles, pain, and fainting.^43,44^ In addition, women are deferred from blood donation because of low hemoglobin levels, iron deficiency anemia, pregnancy, lactation, and low body weight.^43,45^ Interventions that address common fears (needs, anemia, and pain) and misconceptions are central in increasing practice.^11^

The present study estimated that good knowledge (29.08%) was lower than that reported by Samreen et al. (41.9%), Urgesa et al. (43.5%), Beyene et al. (47%), Mussema et al. (48.3%), Jemberu et al. (56.5%) and Nnachi et al. (71.2%) but higher than that reported by Kalyani et al. (19%).^11–13,27,31,34^ These disparities may have arisen because of differences in the study populations, methods of assessment, and categorization of the level of knowledge. Age was observed to be a significant predictor of good knowledge (AOR = 0.951). The association of lower levels of knowledge with increasing age has also been noted in other studies^12,13,34,36^. In addition, being a graduate or above in terms of education was significantly associated with good knowledge (AOR: 5.083). Similar studies from India and Ethiopia have also demonstrated that higher educational levels are related to better knowledge of blood donation.^12,13,46^ Good knowledge regarding blood donation was also observed in those who had donated blood in the past (AOR: 2.808). This may be due to the educational material and counseling that an individual receives during the process of donating blood.

A little over a quarter (26.41%) of the respondents exhibited favorable attitudes toward blood donation. Higher levels of favorable attitudes were reported by Urgesa et al. (32.9%), Beyene et al. (48%), Mussema et al. (49.5%%), Jemberu et al. (52.2%), Samreen et al. (57%), and Nnachi et al. (62.9%).^11–13,27,31,34^ These dissimilarities might also be attributed to differences in study populations, methods of attitude assessment, and categorization of attitude type. Multivariate logistic regression analysis revealed that a prior history of blood donation was significantly associated with a favorable attitude toward blood donation (AOR: 2.599). In addition, possession of a ration card was associated with decreased odds of a favorable attitude (AOR: 0.311). The possession of a ratio card is indicative of a lower socioeconomic status, which could contribute to unfavorable attitudes through its associations with lower educational levels and other adverse cultural factors and beliefs. This study also revealed that the knowledge score was positively associated with favorable attitudes (AOR: 1.221). Higher levels of knowledge translate into better attitudes, a finding also substantiated by Jemberu et al. in their study.^12^ These factors affecting knowledge and attitudes must also be considered, as they directly affect practices while designing programs and strategies to improve voluntary blood donation.

### Strengths and limitations

The present study was conducted at the community level among a rural population, which serves as its major strength. However, as this was a cross-sectional study, inferences of a causal nature cannot be derived from the associations observed. The generalizability of the findings is limited, as the study was conducted in a single location. Moreover, social desirability bias cannot be excluded because of the reliance on self-reported data by the participants. In addition, complete validation of the study tool, especially construct validity, criterion validity, and factor analysis, could not be performed.

## Conclusion and recommendations

In our study, nearly two out of three participants were aware of blood donation, but hardly one out of three was aware of their blood group. Only slightly more than one-fourth had good knowledge and a favorable attitude toward blood donation, while only one out of ten had donated blood in the past. There was no specific reason for not donating blood, followed by dislike or fear of needles, which were the most common barriers to blood donation among the participants. Younger age, an education level of graduate and above, and a history of prior blood donation were possible independent predictors of good knowledge regarding blood donation. Not possessing a ration card, a history of prior blood donation, and high knowledge regarding blood donation may be associated with favorable attitudes toward blood donation.

Development of a targeted educational intervention to improve awareness regarding blood donation and blood groups. Exploring techniques such as relaxation, simulations, and improved donor comfort to reduce needle-related phobia and fear of pain during blood donation can improve donation practices in the community. A further qualitative study exploring the social and cultural factors that influence blood donation would add more information to this topic.

## Supplemental Material

sj-docx-1-smo-10.1177_20503121251387217 – Supplemental material for Knowledge, attitudes, and practices regarding blood donation among rural adults aged 18–59 years in Bihar, India: A community-based cross-sectional study Supplemental material, sj-docx-1-smo-10.1177_20503121251387217 for Knowledge, attitudes, and practices regarding blood donation among rural adults aged 18–59 years in Bihar, India: A community-based cross-sectional study by Manisha Verma, Shreyas Patil, Rajath Rao, Bijaya Nanda Naik, Santosh Kumar Nirala and Mohit Bhardwaj in SAGE Open Medicine

sj-docx-2-smo-10.1177_20503121251387217 – Supplemental material for Knowledge, attitudes, and practices regarding blood donation among rural adults aged 18–59 years in Bihar, India: A community-based cross-sectional study Supplemental material, sj-docx-2-smo-10.1177_20503121251387217 for Knowledge, attitudes, and practices regarding blood donation among rural adults aged 18–59 years in Bihar, India: A community-based cross-sectional study by Manisha Verma, Shreyas Patil, Rajath Rao, Bijaya Nanda Naik, Santosh Kumar Nirala and Mohit Bhardwaj in SAGE Open Medicine
