# Supplementary material for: Knowledge, attitudes, and practices regarding blood donation among rural adults aged 18–59 years in Bihar, India: A community-based cross-sectional study
Source: SAGE Open Med. 2026 Feb 5;14:20503121251387217. doi: 10.1177/20503121251387217 (PMC12876646; doi:10.1177/20503121251387217)
Supplement: sj-docx-1-smo-10.1177_20503121251387217 – Supplemental material for Knowledge, attitudes, and practices regarding blood donation among rural adults aged 18–59 years in Bihar, India: A community-based cross-sectional study [file sj-docx-1-smo-10.1177_20503121251387217.docx]

**Knowledge, attitude and Practices regarding Blood Donation among the rural adults aged 18-59 years in Bihar, India: A community-based cross-sectional study**

**Study population: All adults (18 to 59 years) residing in rural field practice area of AIIMS Patna**

**Section A: Sociodemographic details**

1. SI. No:
2. Date:
3. Name:
4. Age (in years):
5. Sex: Male Female
6. Residence:Urban Rural
7. Religion: Hindu Muslim Sikh Christian Others
8. Education:

Illiterate Primary Secondary High School Graduate

Graduate and above

1. Occupation:
2. Colour of Ration Card:Red Blue Yellow others
3. Blood Group: A+ B+ O+ AB+

A- B- O- AB- Don’t know

1. Type of family:

**Section B: Knowledge regarding blood donation**

1. Have you heard about blood donation? Yes No Not sure

If yes, what is the source?

1. Who can donate blood? ………………. Don’t know
2. How much blood should be donated during one session of blood donation?

ml Don’t know

1. What should be the minimum weight for a person to donate blood?

kg Don’t know

1. What is the minimum time interval between one blood donation and next blood donation? months Don’t Know
2. How many times a person can donate blood in one year? __________________
3. Where can one donate blood?  Don’t know
4. What infections can transmit through blood donation? __________________ Don’t know
5. What is the legal age for blood donation in our country? years

Don’t know

1. Can a person with low Hb/Anaemia donate blood? Yes No

**Section C: Attitude**

1. Do you think that blood donation is an important act? Yes no Don’t know
2. Do you think donating blood can save lives? Yes no Don’t know
3. According to your knowledge, can people with any blood type donate blood?

Yes No Don’t know

1. Will you donate the blood voluntarily? Yes no Don’t know
2. If you agree to donate the blood, where do you prefer to donate?
3. Blood bank
4. Residence
5. Workplace
6. Others
7. Do you agree that donors should be paid to promote blood donation?
8. Yes
9. No
10. Don’t know

**Section D: Practice**

1. Have you donated blood any time in the past? Yes No

If no, skip to section E.

1. If yes, a) how many times have you donated blood? _________________________

b) What was the reason behind your donation?

c) Did you have any health problem after blood donation? Yes No

1. Did you receive any information before blood donation? Yes no

If yes, then what was the mode of information? Only verbal verbal and printed

1. Were there any health-related questions asked before donation? ☐Yes ☐No
2. Were there any investigations done before donation? Yes No

If yes, enumerate them.

1. Did you undergo any medical examination before blood donation? Yes  no

If yes, specify ____________________________________________

1. Was there any refreshment provided after blood donation? Yes No

**Section E: Barriers against blood donation**

1. What are the reasons for not donating/ not willing to donate blood? (open question)

Prompts

- The hours are inconvenient.
- I don’t like needles
- It would be painful
- I would feel faint, dizzy, or nauseated
- I would bruise and my arm would be sore.
- No specific reason.
- Don’t want to donate blood
- There is myth/ superstition that if I donate blood, I will get weak.
- It will be waste of wage, time.
- The process is complicated.
- The blood donation camp staff is not cooperative or of bad behaviour.
- Others
